# Supplementary material for: Body Mass Index Mediates the Relationship between the Frequency of Eating Away from Home and Hypertension in Rural Adults: A Large-Scale Cross-Sectional Study
Source: Nutrients. 2022 Apr 27;14(9):1832. doi: 10.3390/nu14091832 (PMC9104948; doi:10.3390/nu14091832)
Supplement: Supplementary file 1 [file nutrients-14-01832-s001.zip › nutrients-1647198-supplementary.pdf]

Table S1. Multivariate-adjusted *β*-coefficient and 95% *CI* for SBP according to weekly frequency of EAFH.

Weekly frequency of EAFH	SBP		*β* (95% *CI*)				*P_{trend}
	Mean	SD	Model 1	Model 2	Model 3	*Per level risk	
**Total (n = 29,611)**						**0.241 (0.173, 0.308)**	**<0.001**
**0 time (n = 25935)**	126.138	20.274	0 (Ref.)	0 (Ref.)	0 (Ref.)		
**1~2 times (n = 1051)**	121.649	18.313	**1.823 (0.664, 2.982)**	**1.753 (0.590, 2.916)**	**1.336 (0.196, 2.476)**		
**3~4 times (n = 513)**	120.136	16.751	**2.748 (1.107, 4.389)**	**2.678 (1.030, 4.326)**	**2.230 (0.613, 3.846)**		
**5~6 times (n = 345)**	121.290	17.053	**4.423 (2.433, 6.412)**	**4.334 (2.337 ,6.332)**	**3.758 (1.798, 5.719)**		
**≥ 7 times (n = 1,767)**	123.500	17.682	**4.528 (3.611, 5.446)**	**4.486 (3.552, 5.420)**	**3.931 (3.008, 4.853)**		
**Men (n = 12,098)**						**0.187 (0.104, 0.270)**	**< 0.001**
**0 time (n = 9825)**	126.432	18.959	0 (Ref.)	0 (Ref.)	0 (Ref.)		
**1~2 times (n = 523)**	126.637	17.760	**3.261 (1.649, 4.873)**	**3.113 (1.503, 4.724)**	**2.507 (0.936, 4.079)**		
**3~4 times (n = 317)**	123.735	15.745	1.982 (-0.079, 4.042)	1.755 (-0.304, 3.815)	1.505 (-0.502, 3.513)		
**5~6 times (n = 228)**	124.789	15.181	**3.665 (1.249, 6.082)**	**3.310 (0.895, 5.726)**	**2.831 (0.474, 5.187)**		
**≥ 7 times (n = 1,205)**	126.258	16.776	**4.203 (3.063, 5.342)**	**3.917 (2.775, 5.059)**	**3.504 (2.380, 4.627)**		
**Women (n = 17,513)**						0.071 (-0.043, 0.185)	0.222
**0 time (n = 16,110)**	125.958	21.035	0 (Ref.)	0 (Ref.)	0 (Ref.)		
**1~2 times (n = 528)**	116.708	17.510	0.528 (-1.099, 2.155)	0.417 (-1.216, 2.049)	0.435 (-1.172, 2.041)		
**3~4 times (n = 196)**	114.316	16.731	1.931 (-0.702, 4.564)	1.773 (-0.866, 4.413)	1.497 (-1.101, 4.094)		
**5~6 times (n = 117)**	114.470	18.451	1.903 (-1.481, 5.287)	1.868 (-1.524, 5.260)	2.035 (-1.303, 5.374)		
**≥ 7 times (n = 562)**	117.587	18.133	1.325 (-0.254, 2.905)	1.216 (-0.369, 2.801)	1.161 (-0.400, 2.723)		

CI confidence interval; SBP systemic blood pressure; EAFH eating away from home.

*Full-adjusted model for age, gender (only for total participants), marital status, education level, average income per month, smoking status, alcohol consumption, physical activity, abundant vegetable and fruit intake, high fat diet and family history of hypertension.

Model 1: adjusted for age;

Model 2: adjusted for age, gender (only for total participants), marital status, education level, average income per month;

Model 3: adjusted for age, gender (only for total participants), marital status, education level, average income per month, smoking status, alcohol consumption, physical activity, abundant vegetable and fruit intake, high fat diet and family history of hypertension.

Trends of odds ratios were performed using the categories (0time, 1~2 times, 3~4 times, 5~6 times, ≥ 7 times) of weekly frequency of EAFH group as a continuous variable in the logistic regression model.

Table S2. Multivariate-adjusted *β*-coefficient and 95% *CI* for DBP according to weekly frequency of EAFH.

**Weekly frequency of EAFH**	**DBP**		***β* (95% *CI*)**				***P* trend**
	**Mean**	**SD**	**Model 1**	**Model 2**	**Model 3**	***Per level risk**	
**Total (n = 29,611)**						**0.174 (0.132, 0.216)**	**<0.001**
**0 time (n = 25935)**	77.205	11.597	0 (Ref.)	0 (Ref.)	0 (Ref.)		
**1~2 times (n = 1051)**	77.814	11.685	**1.573 (0.850, 2.297)**	**1.360 (0.636, 2.084)**	**0.999 (0.291, 1.706)**		
**3~4 times (n = 513)**	77.957	11.783	**2.089 (1.065, 3.114)**	**1.693 (0.667, 2.720)**	**1.308 (0.304, 2.311)**		
**5~6 times (n = 345)**	79.009	11.637	**3.221 (1.979, 4.463)**	**2.811 (1.567, 4.055)**	**2.296 (1.079, 3.513)**		
**≥ 7 times (n = 1,767)**	79.895	12.235	**3.785 (3.212, 4.358)**	**3.293 (2.711, 3.874)**	**2.825 (2.252, 3.398)**		
**Men (n = 12,098)**						**0.166 (0.112, 0.220)**	**< 0.001**
**0 time (n = 9825)**	77.848	11.837	0 (Ref.)	0 (Ref.)	0 (Ref.)		
**1~2 times (n = 523)**	80.532	12.091	**2.386 (1.330, 3.442)**	**2.285 (1.233, 3.338)**	**1.709 (0.690, 2.727)**		
**3~4 times (n = 317)**	80.129	11.502	**1.825 (0.475, 3.175)**	**1.642 (0.297, 2.988)**	**1.314 (0.013, 2.615)**		
**5~6 times (n = 228)**	81.421	11.449	**3.056 (1.473, 4.639)**	**2.861 (1.283, 4.440)**	**2.356 (0.829, 3.884)**		
**≥ 7 times (n = 1,205)**	81.921	12.274	**3.647 (2.900, 4.393)**	**3.430 (2.684, 4.177)**	**2.990 (2.262, 3.718)**		
**Women (n = 17,513)**						0.063 (-0.006, 0.132)	0.075
**0 time (n = 16,110)**	76.814	11.431	0 (Ref.)	0 (Ref.)	0 (Ref.)		
**1~2 times (n = 528)**	75.121	10.614	0.330 (-0.657, 1.317)	0.518 (-0.472, 1.507)	0.506 (-0.469, 1.480)		
**3~4 times (n = 196)**	74.444	11.406	0.437 (-1.160, 2.034)	0.755 (-0.844, 2.355)	0.530 (-1.046, 2.106)		
**5~6 times (n = 117)**	74.308	10.551	0.263 (-1.789, 2.316)	0.769 (-1.286, 2.825)	0.754 (-1.271, 2.780)		
**≥ 7 times (n = 562)**	75.550	10.962	0.741 (-0.217, 1.700)	0.954 (-0.007, 1.914)	0.880 (-0.067, 1.827)		

CI confidence interval; DBP diastolic blood pressure; EAFH eating away from home.

*Full-adjusted model for age, gender (only for total participants), marital status, education level, average income per month, smoking status, alcohol consumption, physical activity, abundant vegetable and fruit intake, high fat diet and family history of hypertension.

Model 1: adjusted for age;

Model 2: adjusted for age, gender (only for total participants), marital status, education level, average income per month;

Model 3: adjusted for age, gender (only for total participants), marital status, education level, average income per month, smoking status, alcohol consumption, physical activity, abundant vegetable and fruit intake, high fat diet and family history of hypertension.

Trends of odds ratios were performed using the categories (0time, 1~2 times, 3~4 times, 5~6 times, ≥ 7 times) of weekly frequency of EAFH group as a continuous variable in the logistic regression model.

Table S3. Multivariate-adjusted *OR* and 95% *CI* for hypertension according to weekly frequency of EAFH breakfasts.

Weekly frequency of EAFH	Prevalence, % (95% *CI*)	*OR* (95% *CI*)				†P_{trend}
		Model 1	Model 2	Model 3	†Per level risk	
**Total (n = 29,611)**					**1.055 (1.029, 1.081)**	**< 0.001**
**0 time (n= 28042)**	32.37 (31.82, 32.92)	1 (Ref.)	1 (Ref.)	1 (Ref.)		
**1~2 times (n= 491)**	23.42 (19.66, 27.18)	**1.397 (1.118, 1.745)**	**1.421 (1.136, 1.777)**	**1.447 (1.149, 1.823)**		
**3~4 times (n= 311)**	22.19 (17.54, 26.83)	1.242 (0.938, 1.644)	1.280 (0.966, 1.697)	1.267 (0.946, 1.697)		
**5~6 times (n= 154)**	28.57 (21.36, 35.79)	**1.683 (1.167, 2.428)**	**1.730 (1.197, 2.501)**	**1.830 (1.249, 2.682)**		
**7 times (n = 613)**	29.36 (25.75, 32.98)	**1.295 (1.077, 1.557)**	**1.340 (1.113, 1.613)**	**1.329 (1.097, 1.611)**		
**Men (n = 12,098)**					**1.043 (1.013, 1.075)**	**0.005**
**0 time (n = 11,118)**	32.32 (31.45, 33.19)	1 (Ref.)	1 (Ref.)	1 (Ref.)		
**1~2 times (n = 270)**	30.37 (24.85, 35.89)	**1.458 (1.111, 1.913)**	**1.419 (1.081, 1.864)**	**1.473 (1.109, 1.957)**		
**3~4 times (n = 212)**	27.83 (21.75, 33.91)	1.223 (0.897, 1.668)	1.197 (0.877, 1.634)	1.223 (0.883, 1.693)		
**5~6 times (n = 110)**	31.82 (22.98, 40.66)	1.488 (0.985, 2.246)	1.438 (0.951, 2.174)	**1.565 (1.013, 2.418)**		
**7 times (n = 388)**	31.70 (27.05, 36.35)	**1.257 (1.007, 1.571)**	1.247 (0.997, 1.559)	1.259 (0.997, 1.589)		
**Women (n = 17,513)**					1.033 (0.988, 1.081)	0.154
**0 time (n = 16,924)**	32.40 (31.70, 33.11)	1 (Ref.)	1 (Ref.)	1 (Ref.)		
**1~2 times (n = 221)**	14.93 (10.20, 19.67)	1.057 (0.709, 1.575)	1.111 (0.743, 1.661)	1.166 (0.773, 1.760)		
**3~4 times (n = 99)**	10.10 (4.06, 16.14)	0.798 (0.403, 1.579)	0.825 (0.415, 1.639)	0.793 (0.394, 1.595)		
**5~6 times (n = 44)**	20.45 (8.05, 32.86)	1.587 (0.731, 3.446)	1.730 (0.788, 3.797)	1.890 (0.848, 4.209)		
**7 times (n = 225)**	25.33 (19.61, 31.06)	1.203 (0.866, 1.671)	1.241 (0.891, 1.727)	1.210 (0.859, 1.704)		

CI confidence interval; *OR* odds ratio; EAFH eating away from home.

*Full-adjusted model for age, gender (only for total participants), marital status, education level, average income per month, smoking status, alcohol consumption, physical activity, abundant vegetable and fruit intake, high fat diet and family history of hypertension.

Model 1: adjusted for age;

Model 2: adjusted for age, gender (only for total participants), marital status, education level, average income per month;

Model 3: adjusted for age, gender (only for total participants), marital status, education level, average income per month, smoking status, alcohol consumption, physical activity, abundant vegetable and fruit intake, high fat diet and family history of hypertension.

Trends of odds ratios were performed using the categories (0time, 1~2 times, 3~4 times, 5~6 times, ≥ 7 times) of weekly frequency of EAFH group as a continuous variable in the logistic regression model.

Table S4. Multivariate-adjusted *OR* and 95% *CI* for hypertension according to weekly frequency of EAFH lunches.

Weekly frequency of EAFH	Prevalence, % (95% *CI*)	*OR* (95% *CI*)				†P_{trend}
		Model 1	Model 2	Model 3	†Per level risk	
**Total (n = 29,611)**					**1.075 (1.054, 1.096)**	**< 0.001**
**0 time (n = 26928)**	32.47 (31.91, 33.03)	1 (Ref.)	1 (Ref.)	1 (Ref.)		
**1~2 times (n = 864)**	25.23 (22.33, 28.13)	**1.236 (1.049, 1.457)**	**1.277 (1.082, 1.506)**	**1.252 (1.055, 1.486)**		
**3~4 times (n =451)**	28.38 (24.20, 32.56)	**1.545 (1.244, 1.919)**	**1.601 (1.287, 1.992)**	**1.534 (1.224, 1.924)**		
**5~6 times (n = 285)**	28.07 (22.82, 33.32)	**1.819 (1.386, 2.387)**	**1.930 (1.467, 2.537)**	**1.898 (1.429, 2.521)**		
**7 times (n = 1,083)**	29.18 (26.47, 31.89)	**1.522 (1.321, 1.753)**	**1.576 (1.366, 1.818)**	**1.541 (1.329, 1.787)**		
**Men (n = 12,098)**					**1.074 (1.049, 1.099)**	**< 0.001**
**0 time (n = 10,345)**	32.04 (31.15, 32.94)	1 (Ref.)	1 (Ref.)	1 (Ref.)		
**1~2 times (n = 517)**	31.53 (27.51, 35.55)	**1.414 (1.161, 1.722)**	**1.388 (1.139, 1.691)**	**1.393 (1.134, 1.712)**		
**3~4 times (n = 330)**	33.94 (28.80, 39.07)	**1.642 (1.293, 2.087)**	**1.580 (1.242, 2.009)**	**1.519 (1.182, 1.952)**		
**5~6 times (n = 209)**	34.45 (27.95, 40.95)	**1.885 (1.400, 2.540)**	**1.846 (1.369, 2.489)**	**1.798 (1.315, 2.458)**		
**7 times (n = 697)**	33.00 (29.50, 36.50)	**1.555 (1.311, 1.845)**	**1.522 (1.282, 1.806)**	**1.536 (1.284, 1.838)**		
**Women (n = 17,513)**					1.017 (0.980, 1.055)	0.374
**0 time (n = 16,583)**	32.73 (32.02, 33.45)	1 (Ref.)	1 (Ref.)	1 (Ref.)		
**1~2 times (n = 347)**	15.85 (11.99, 19.71)	0.833 (0.611, 1.134)	0.861 (0.631, 1.175)	0.849 (0.616, 1.171)		
**3~4 times (n = 121)**	13.22 (7.10, 19.35)	0.827 (0.478, 1.433)	0.847 (0.488, 1.471)	0.892 (0.507, 1.569)		
**5~6 times (n = 76)**	10.53 (3.47, 17.59)	0.779 (0.367, 1.656)	0.828 (0.387, 1.768)	0.924 (0.426, 2.004)		
**7 times (n = 386)**	22.28 (18.11, 26.45)	1.221 (0.941, 1.584)	1.238 (0.953, 1.608)	1.192 (0.910,1.560)		

CI confidence interval; *OR* odds ratio; EAFH eating away from home.

*Full-adjusted model for age, gender (only for total participants), marital status, education level, average income per month, smoking status, alcohol consumption, physical activity, abundant vegetable and fruit intake, high fat diet and family history of hypertension.

Model 1: adjusted for age;

Model 2: adjusted for age, gender (only for total participants), marital status, education level, average income per month;

Model 3: adjusted for age, gender (only for total participants), marital status, education level, average income per month, smoking status, alcohol consumption, physical activity, abundant vegetable and fruit intake, high fat diet and family history of hypertension.

Trends of odds ratios were performed using the categories (0time, 1~2 times, 3~4 times, 5~6 times, ≥ 7 times) of weekly frequency of EAFH group as a continuous variable in the logistic regression model.

Table S5. Multivariate-adjusted *OR* and 95% *CI* for hypertension according to weekly frequency of EAFH dinners.

Weekly frequency of EAFH	Prevalence, % (95% *CI*)	*OR* (95% *CI*)				†P_{trend}
		Model 1	Model 2	Model 3	†Per level risk	
**Total (n = 29,611)**					**1.066 (1.040, 1.093)**	**< 0.001**
**0 time (n = 27541)**	32.51 (31.96, 33.06)	1 (Ref.)	1 (Ref.)	1 (Ref.)		
**1~2 times (n = 843)**	24.20 (21.30, 27.10)	**1.480 (1.249, 1.753)**	**1.512 (1.274, 1.794)**	**1.410 (1.180, 1.685)**		
**3~4 times (n = 413)**	28.57 (24.20, 32.95)	**1.921 (1.532, 2.408)**	**2.009 (1.598, 2.526)**	**1.913 (1.510, 2.426)**		
**5~6 times (n = 216)**	25.46 (19.61, 31.32)	**1.675 (1.214, 2.311)**	**1.751 (1.266, 2.422)**	**1.866 (1.331, 2.616)**		
**7 times (n = 598)**	25.75 (22.24, 29.27)	**1.234 (1.016, 1.500)**	**1.291 (1.061, 1.571)**	**1.268 (1.035, 1.553)**		
**Men (n = 12,098)**					**1.044 (1.013, 1.075)**	**0.004**
**0 time (n = 10,721)**	32.39 (31.51, 33.28)	1 (Ref.)	1 (Ref.)	1 (Ref.)		
**1~2 times (n = 488)**	31.35 (27.22, 35.48)	**1.596 (1.300, 1.960)**	**1.553 (1.264, 1.909)**	**1.400 (1.128, 1.738)**		
**3~4 times (n = 325)**	34.77 (29.56, 39.97)	**1.910 (1.500, 2.433)**	**1.820 (1.426, 2.321)**	**1.713 (1.328, 2.208)**		
**5~6 times (n = 170)**	28.24 (21.40, 35.07)	**1.508 (1.066, 2.134)**	**1.462 (1.032, 2.070)**	**1.573 (1.094, 2.263)**		
**7 times (n = 394)**	26.65 (22.27, 31.03)	1.134 (0.897, 1.433)	1.122 (0.887, 1.420)	1.110 (0.869, 1.418)		
**Women (n = 17,513)**					1.017 (0.969, 1.067)	0.492
**0 time (n = 16,820)**	32.59 (31.88, 33.29)	1 (Ref.)	1 (Ref.)	1 (Ref.)		
**1~2 times (n = 355)**	14.37 (10.70, 18.03)	0.975 (0.707, 1.346)	1.000 (0.723, 1.385)	1.037 (0.741, 1.452)		
**3~4 times (n = 88)**	5.68 (0.75, 10.61)	0.433 (0.172, 1.093)	0.458 (0.181, 1.159	0.518 (0.202, 1.327)		
**5~6 times (n = 46)**	15.22 (4.43, 26.00)	0.851 (0.357, 2.032)	0.853 (0.356, 2.047)	1.015 (0.411, 2.505)		
**7 times (n = 204)**	24.02 (18.11, 29.93)	1.192 (0.841, 1.689)	1.217 (0.858, 1.726)	1.187 (0.827, 1.703)		

CI confidence interval; *OR* odds ratio; EAFH eating away from home.

*Full-adjusted model for age, gender (only for total participants), marital status, education level, average income per month, smoking status, alcohol consumption, physical activity, abundant vegetable and fruit intake, high fat diet and family history of hypertension.

Model 1: adjusted for age;

Model 2: adjusted for age, gender (only for total participants), marital status, education level, average income per month;

Model 3: adjusted for age, gender (only for total participants), marital status, education level, average income per month, smoking status, alcohol consumption, physical activity, abundant vegetable and fruit intake, high fat diet and family history of hypertension.

Trends of odds ratios were performed using the categories (0time, 1~2 times, 3~4 times, 5~6 times, ≥ 7 times) of weekly frequency of EAFH group as a continuous variable in the logistic regression model.

Table S6. Multivariate-adjusted *β*-coefficient and 95% *CI* for BMI according to weekly frequency of EAFH.

Weekly frequency of EAFH	BMI		*β* (95% *CI*)				*P_{trend}
	Mean	SD	Model 1	Model 2	Model 3	*Per level risk	
**Total (n = 29,611)**						**0.036 (0.023, 0.050)**	**<0.001**
**0 time (n = 25935)**	24.676	3.547	0 (Ref.)	0 (Ref.)	0 (Ref.)		
**1~2 times (n = 1051)**	25.019	3.698	**0.298 (0.075, 0.520)**	**0.416 (0.194, 0.639)**	**0.331 (0.111, 0.551)**		
**3~4 times (n = 513)**	25.209	3.734	**0.470 (0.156, 0.785)**	**0.660 (0.346, 0.975)**	**0.569 (0.258, 0.881)**		
**5~6 times (n = 345)**	25.323	3.618	**0.580 (0.198, 0.962)**	**0.821 (0.440, 1.203)**	**0.703 (0.324, 1.082)**		
**≥ 7 times (n = 1,767)**	25.168	3.553	**0.440 (0.264, 0.616)**	**0.643 (0.465, 0.822)**	**0.540 (0.362, 0.718)**		
**Men (n = 12,098)**						**0.034 (0.019, 0.050)**	**< 0.001**
**0 time (n = 9825)**	24.270	3.403	0 (Ref.)	0 (Ref.)	0 (Ref.)		
**1~2 times (n = 523)**	25.460	3.545	**0.892 (0.588, 1.196)**	**0.845 (0.544, 1.147)**	**0.721 (0.425, 1.018)**		
**3~4 times (n = 317)**	25.714	3.624	**0.989 (0.601, 1.377)**	**0.908 (0.523, 1.293)**	**0.846 (0.467, 1.224)**		
**5~6 times (n = 228)**	25.610	3.648	**0.824 (0.369, 1.280)**	**0.763 (0.311, 1.216)**	**0.679 (0.234, 1.124)**		
**≥ 7 times (n = 1,205)**	25.436	3.525	**0.740 (0.526, 0.955)**	**0.655 (0.442, 0.869)**	**0.553 (0.341, 0.765)**		
**Women (n = 17,513)**						0.000 (-0.022, 0.023)	0.975
**0 time (n = 16,110)**	24.923	3.610	0 (Ref.)	0 (Ref.)	0 (Ref.)		
**1~2 times (n = 528)**	24.584	3.796	-0.122 (-0.440, 0.196)	0.039 (-0.279, 0.357)	0.022 (-0.294, 0.339)		
**3~4 times (n = 196)**	24.393	3.774	-0.228 (-0.742, 0.286)	0.009 (-0.504, 0.523)	-0.062 (-0.573, 0.450)		
**5~6 times (n = 117)**	24.766	3.507	0.141 (-0.519, 0.801)	0.425 (-0.235, 1.085)	0.371 (-0.287, 1.028)		
**≥ 7 times (n = 562)**	24.592	3.547	-0.115 (-0.423, 0.193)	0.048 (-0.261, 0.356)	0.030 (-0.277, 0.338)		

CI confidence interval; BMI body mass index; EAFH eating away from home.

*Full-adjusted model for age, gender (only for total participants), marital status, education level, average income per month, smoking status, alcohol consumption, physical activity, abundant vegetable and fruit intake, high fat diet and family history of hypertension.

Model 1: adjusted for age;

Model 2: adjusted for age, gender (only for total participants), marital status, education level, average income per month;

Model 3: adjusted for age, gender (only for total participants), marital status, education level, average income per month, smoking status, alcohol consumption, physical activity, abundant vegetable and fruit intake, high fat diet and family history of hypertension.

Trends of odds ratios were performed using the categories (0time, 1~2 times, 3~4 times, 5~6 times, ≥ 7 times) of weekly frequency of EAFH group as a continuous variable in the logistic regression model.

Table S7. Mediation analysis of the relationship between frequency of EAFH and hypertension by BMI

Mediation analysis	Total		Men	
	Parameter estimate (95% *CI*)	*OR* (95% *CI*)	Parameter estimate (95% *CI*)	*OR* (95% *CI*)
**BMI**				
**Total effect**	**0.0301 (0.0218, 0.0392)**	**1.031 (1.022, 1.040)**	**0.0260 (0.0159, 0.0363)**	**1.026 (1.016, 1.037)**
**Direct effect path c′**	**0.0255 (0.0164, 0.0347)**	**1.026 (1.017, 1.035)**	**0.0210 (0.0098, 0.0321)**	**1.021 (1.010, 1.033)**
**Path a**	**0.0361 (0.0230, 0.0493)**	**1.037 (1.023, 1.051)**	**0.0338 (0.0181, 0.0495)**	**1.034 (1.018, 1.051)**
**Path b**	**0.0255 (0.0164, 0.0347)**	**1.026 (1.017, 1.035)**	**0.0210 (0.0098, 0.0321)**	**1.021 (1.010, 1.033)**
**Indirect effect path ab**	**0.0064 (0.0039, 0.0086)**	**1.006 (1.004, 1.009)**	**0.0066 (0.0033, 0.0098)**	**1.007 (1.003, 1.010)**
**Indirect/total effect (%)**	**21.3**		**25.4**	

Adjusted for age, gender (only for total participants), marital status, education level, average income per month, smoking status, alcohol consumption, physical activity, abundant vegetable and fruit intake, high fat diet and family history of hypertension.

Path a indicates the path from the frequency of EAFH to BMI (Mediator).

Path b indicates the path from BMI to hypertension.

Path ab indicates the indirect effect of frequency of EAFH on hypertension mediated by BMI.

Path c′ indicates the direct effect of frequency of EAFH on hypertension.

EAFH eating away from home; BMI body mass index; *CI* confidence interval; *OR* odds ratio.

Table S8. The characteristics of three meals EAFH by gender

Variables	Total (N = 29,611)			Men (N = 12,098)			Women (N = 17,513)		
	Normotensive	Hypertension	P	Normotensive	Hypertension	P	Normotensive	Hypertension	P
**Breakfast (times/week), n (%)**			<0.001			0.665			<0.001
0	18965 (94.2)	9077 (95.7)		7525 (91.7)	3593 (92.3)		11440 (96.0)	5484 (98.0)	
1~2	376 (1.9)	115 (1.2)		188 (2.3)	82 (2.1)		188 (1.6)	33 (0.6)	
3~4	242 (1.2)	69 (0.7)		153 (1.9)	59 (1.5)		89 (0.7)	10 (0.2)	
5~6	110 (0.5)	44 (0.5)		75 (0.9)	35 (0.9)		35 (0.3)	9 (0.2)	
7	433 (2.2)	180 (1.9)		265 (3.2)	123 (3.2)		168 (1.4)	57 (1.0)	
**Lunch (times/week), n (%)**			<0.001			0.851			<0.001
0	18185 (90.4)	8743 (92.2)		7030 (85.7)	3315 (85.2)		11155 (93.6)	5428 (97.0)	
1~2	646 (3.2)	218 (2.3)		354 (4.3)	163 (4.2)		292 (2.4)	55 (1.0)	
3~4	323 (1.6)	128 (1.3)		218 (2.7)	112 (2.9)		105 (0.9)	16 (0.3)	
5~6	205 (1.0)	80 (0.8)		137 (1.7)	72 (1.8)		68 (0.6)	8 (0.1)	
7	767 (3.8)	316 (3.4)		467 (5.6)	230 (5.9)		300 (2.5)	86 (1.6)	
**Dinner (times/week), n (%)**			<0.001			0.088			<0.001
0	18587 (92.4)	8954 (94.4)		7248 (88.3)	3473 (89.2)		11339 (95.1)	5481 (98.0)	
1~2	639 (3.2)	204 (2.2)		335 (4.1)	153 (3.9)		304 (2.6)	51 (0.9)	
3~4	295 (1.5)	118 (1.2)		212 (2.6)	113 (2.9)		83 (0.7)	5 (0.1)	
5~6	161 (0.8)	55 (0.6)		122 (1.5)	48 (1.2)		39 (0.3)	7 (0.1)	
7	444 (2.1)	154 (1.6)		289 (3.5)	105 (2.8)		155 (1.3)	49 (0.9)	

Categorical variables are expressed as percentages. EAFH, eating away from home.
